# Assessment of Potentially Toxic Metals in Fish from Lake Manyara, Northern Tanzania

**DOI:** 10.1007/s00128-023-03794-6

**Published:** 2023-09-13

**Authors:** Shovi Sawe, Aloyce Amasi, Maarten Wynants

**Affiliations:** 1 Department of Research and Development, Tanzania Atomic Energy Commission, P O Box 743, Arusha, Tanzania; 2 School of Materials, Energy, Water and Environmental Science, The Nelson Mandela African Institution of Science and Technology, P.O. Box 447, 23311 Arusha, Tanzania; 3 Department of Soil and Environment, Swedish University of Agricultural Sciences, Lennart Hjelms väg 9, 75007 Uppsala, Sweden

**Keywords:** East African Rift lakes, Energy dispersive X-ray fluorescence, Recommended weekly intake, Permissible levels, Zinc, Copper, Nikkel, Lead, *Clarias gariepinus*, *Oreochromis amphimelas*

## Abstract

Elemental tracer concentrations of copper, lead, nickel and zinc, were assessed in the muscles of *Oreochromis amphimelas* and *Clarias gariepinus* from Lake Manyara, Tanzania, to evaluate their safety to consumers, specifically humans. Results revealed that no elemental concentrations exceeded the FAO permissible levels, indicating fish from all sites are safe for human consumption. However, based on the highest found concentration of Pb, we recommend a maximum consumption of 2.2 kg of fish from Lake Manyara per week. No significant differences were observed in the metal concentrations between the two fish species, suggesting there is no bioaccumulation in the food chain. Moreover, no significant differences were found between fish landing sites, indicating there are no regions in the lake with higher pollution. These findings indicate that PTM concentrations have not increased to toxic levels due to increased mobilisation from the catchment. Continued monitoring of potential toxic metal concentrations in fish is recommended due to endorheic nature of Lake Manyara and increasing anthropogenic activities in its catchment area.

According to URT 2010 and FAO 2020, the world’s fisheries play a significant economic role in the production of food, jobs creation, income, and tax revenue. Globally, around 60 million people are employed in fishing, with the majority in developing nations (FAO 2018). In addition to these economic advantages, fish is important as a source of protein, essential minor nutrients, and nutritive non-saturated fatty acids and Omega-3 that aid in lowering blood cholesterol and preventing heart problems (Erkkilä et al. 2004). However, spills and discharges from industrial, agricultural, and urban sources that contaminated soils, groundwater, and surface water in recent decades have had an adverse effect on most aquatic ecosystems. Heavy metals like cadmium, mercury, lead, zinc and copper are of particular concern because of their toxicity, persistence in the environment, and tendency to accumulate in food chains (Tam and Wong 2000). Fish are considered to be good indicators of water pollution by metals and the degree of biological effects to humans, aquatic species, and the associated food web (Klaverkamp et al. 1984). Metal pollution can impact societies and ecosystems through direct toxicity to humans and aquatic life, as well as indirect toxicity through accumulations of metals in the aquatic food web. The Lake Manyara basin, which includes Lake Manyara National Park, is of great ecological and socio-economic value. The diversity of ecosystems and high biodiversity support agriculture, fisheries, pastoralism, and ecotourism (Janssens de Bisthoven et al. 2020). However, the lake itself is threatened by rising anthropogenic pressures in its watershed. Due to the fact that Lake Manyara is an endorheic system without an outflow, pollution loading poses a particular risk. Agricultural areas have increased from about 10 to 25% over the last three decades, which has increased the basin’s potential for erosion (Wynants et al. 2018). Furthermore, agro-pastoral systems are degrading as a result of unsustainable agricultural methods and overgrazing (Wynants et al. 2021b). The rapid downstream movement of eroded sediments through intricate networks of ephemeral gullies has caused Lake Manyara’s sedimentation rates to quadruple (Wynants et al. 2020). In coffee plantation soils, copper (Cu) concentrations have been found to be relatively higher (Wynants et al. 2021a), which is most likely a result of the use of Cu-based fungicides. Additionally, the Minjingu mine, one of Eastern Africa’s most significant phosphate mines, is situated about 5 km southeast of the lake, raising the possibility of phosphate runoff and other harmful byproducts (Banzi et al. 2000).

Although the effects of eutrophication and toxic algal blooms on the ecosystem of Lake Manyara have already been thoroughly studied (Nonga et al. 2011), it is unknown how potentially toxic metals (PTMs) accumulate in fish. As been shown by Mng’ong’o et al., (2021), PTM concentrations in other Tanzanian water bodies have increased in recent decades as a result of their connection to eroded sediments, direct runoff of agricultural additives, or discharge from mines and sewage treatment plants. Since the presence of commercial and subsistence fisheries in Lake Manyara, it is therefore crucial to look into the levels of PTMs in fish from Lake Manyara in order to protect the health of consumers due to the negative health effects that eating polluted fish may have. The endemic Manyara tilapia (*Oreochromis amphimelas*) from Lake Manyara and the African catfish (*Clarias gariepinus*) were the subjects of this study, in which we examined the levels of PTMs in edible parts of both species. Manyara tilapia are microphagous (Shechonge et al. 2019), while African catfish are omnivores that become more predatory when they mature (Lemmens et al. 2017). In this study, one non-essential element (lead) and three micronutrients (copper, nickel, and zinc) were examined for screening purposes. In the crust of the earth, copper (Cu) naturally occurs as oxides and sulfides, and occasionally as metallic copper. Cu is related to other metals like cadmium, silver, tin, and zinc. It is a micronutrient that many different enzymes and other cell components need in very small concentrations to support essential processes in all living things. Excessive intakes however, can result in gastrointestinal symptoms and liver damage. (Demirezen and Uruc 2006; Ivo et al. 2013; Lee et al. 2021). Natural occurrences of lead (Pb) in the crust of the earth typically occur as a significant component of minerals. However, refinery of Pb and its global use has resulted in significant environmental contamination and health issues due to its high toxicity when concentrated (D’Souza et al. 2011, Mărginean et al. 2016). Pb negatively affects majority of the body’s major organ systems, especially the hematopoietic, renal, nervous, and cardiovascular systems, and even small concentrations of Pb have been shown to cause irreversible health effects. Pb also has no known biological function in the body (Flora et al. 2012). With a content of about 0.008%, nickel (Ni) naturally occurs in the earth’s crust. Similar to Cu, Ni is a micronutrient that helps all living things perform essential tasks, but too high intakes can have adverse health effects, such as teratogenic and genotoxic effects (Demirezen and Uruc 2006; Picarelli et al. 2021). Natural mineral forms of zinc (Zn) are present. According to Tziouvalekas and Karyotis (2018) and Wolf et al. (2022), the largest single source of zinc entering the aquatic environment is the erosion of soil particles containing zinc. Zinc is a crucial trace element known to be a component of several enzyme systems in organisms. However, large quantities of zinc in aquatic ecosystem, can be toxic leading to harmful and undesirable health effects to humans (Li 2019).

The overall aim of this study is to assess if PTMs in fish tissue have increased to harmful concentrations for human consumption. In this context, we hypothesise that concentrations of PTMs in fish tissue would be significantly higher in fish landing sites near river inlets or mining sites where pollution would enter the lake. Moreover, we hypothesise that if PTMs bioaccummulate, their concentration would be higher in the predatory *Clarias gariepinus.*

## Materials and Methods

Lake Manyara is located about 960 m above sea level. It is a shallow alkaline lake formed in a depression in the Rift Valley System of northern Tanzania (Loth and Prins 1986). The lake’s area depth varies significantly depending on the season. The lake reaches its maximum capacity during rainy season when it is 40 km long, 15 km wide, and has a depth of 3.7 m, maximum. The three major contributing rivers are the permanent Dudumera River, originating from the southern highlands, the ephemeral Makuyuni River, originating from the semi-arid savannah and highlands to the east, and the permanent Mto wa Mbu River, originating from the Ngorogoro highland to the north. Intensive irrigation agricultural activities are performed on the floodplains of the Dudumera and Mto wa Mbu rivers near their inlets. The Dudumera and Makuyuni rivers are the major sources of sediment to Lake Manyara (Wynants et al. 2020). Agriculture and mining are some of the human activities in the catchment leading to soil erosion and discharge of pollutants to the lake (Janssens de Bisthoven et al. 2020). Lake Manyara is a soda lake with no outflow and its waters are caustic and saline depending on varying freshwater input during rainy and dry seasons. The aquatic ecology of the lake is characterised by a low diversity of halophilic species, wherein the primary production is typically dominated by the cyanobacterium *Arthrospira fusiformis* (Kihwele et al. 2014). The lake only contains two fish species, African catfish (*Clarias gariepinus*) and endemic Manyara tilapia (*Oreochromis amphimelas*)*.* The fish production in the main lake follows a boom and bust cycle, where productivity halts during the dry season when the waters become too saline and caustic. During these times, the fish population concentrates near the permanent freshwater river inlets, where the salinity and alkalinity is lower. During the wet season, the main lake becomes less caustic and highly productive, and the fish population rapidly booms. *Oreochromis amphimelas* is endemic to Lake Manyara and some other soda lakes of the Tanzanian Rift Valley. It is a maternal mouthbrooder and its population booms during the wet season when the salinity and alkalinity of the lakes goes down. In Lake Manyara, it mainly feeds on the plankton cyanobacteria and other microalgae species (Trewavas 1983). *Clarias gariepinus* is an omnivore and moves down from the permanent freshwater rivers and wetlands to the lake during the wet season to feed on the high number of tilapia. The lake and its surrounding ecosystem supports one of the largest colony of Lesser Flamingo (*Phoeniconaias minor*) and more than 390 other species of birds. During the fishing season (April to November), temporal fishing camps are set up along the northern, eastern, and southwestern shores of the lake, supporting both local households and migratory fishermen. Fishing is mostly concentrated on the eastern and southern parts and is not allowed within roughly one third of the lake that is part of the national park.

**Fig. 1:**
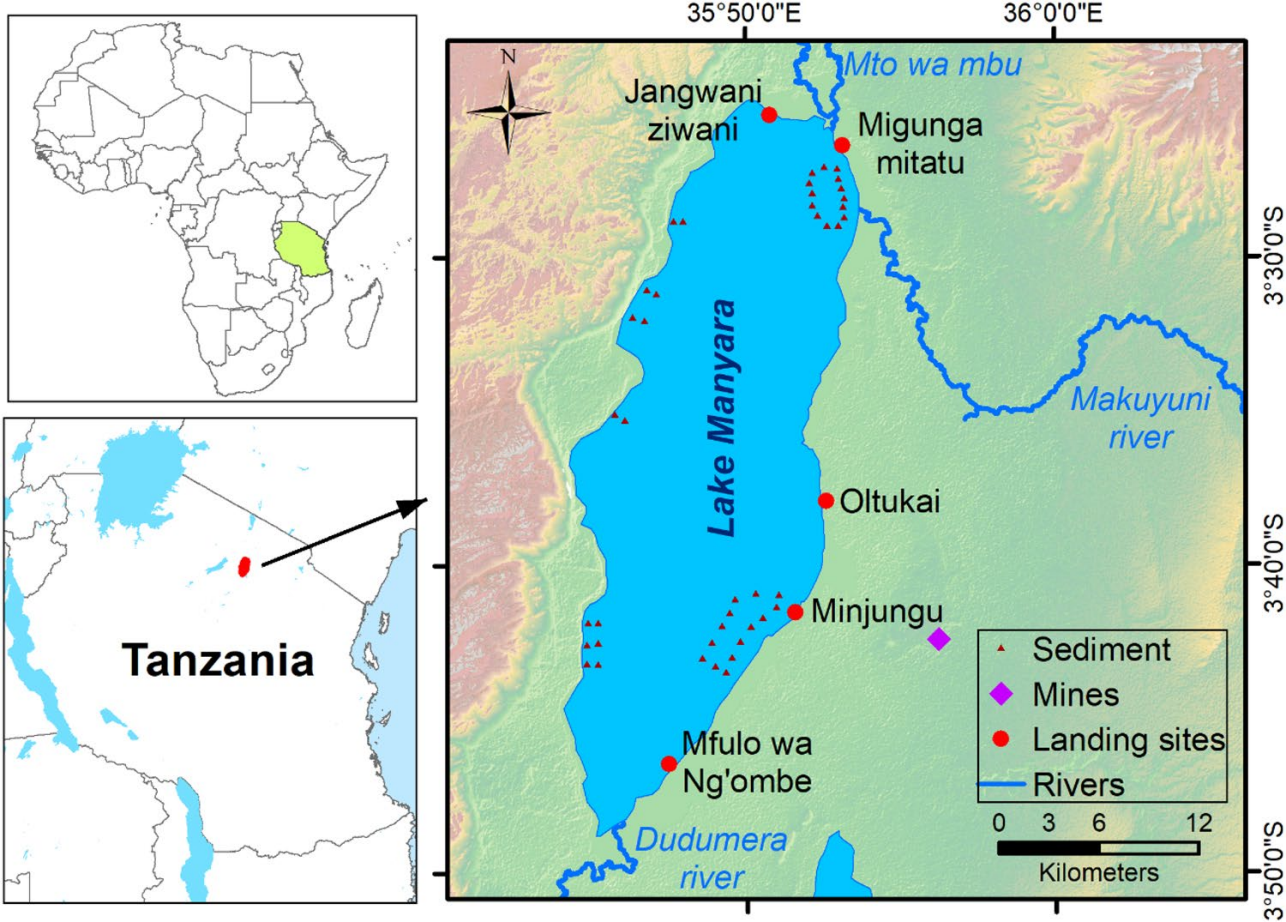
Location of Lake Manyara, fish landing sites, sediment samples, and mines

**Fig. 2:**
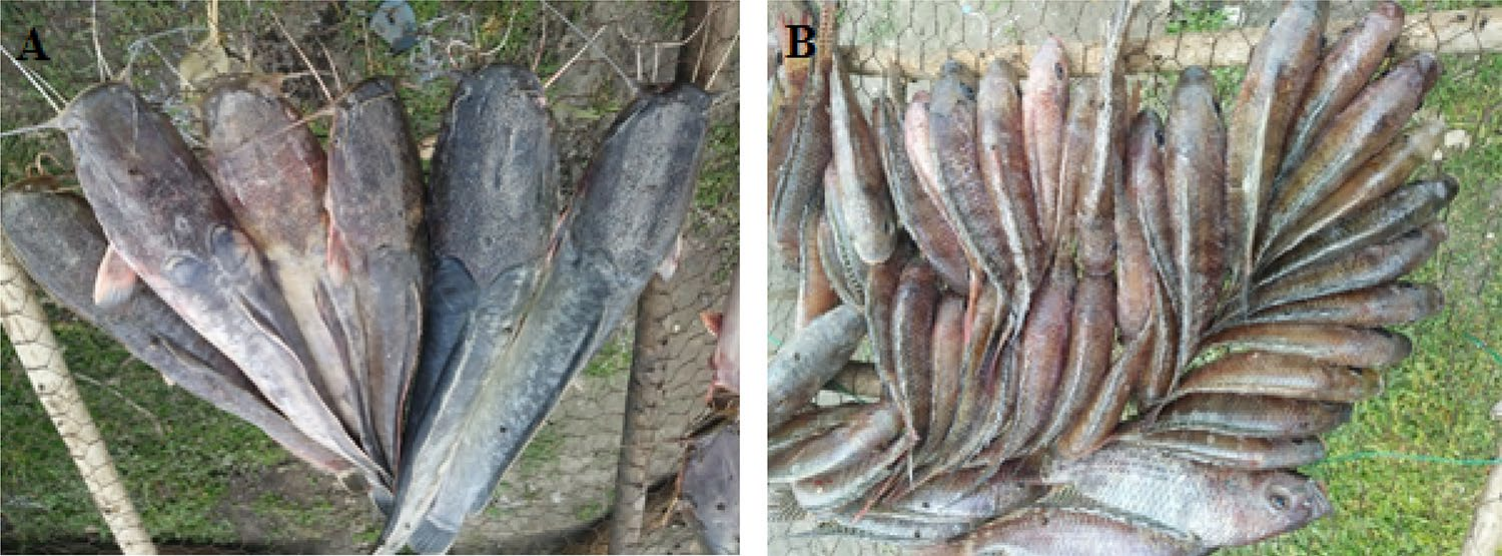
*Clarias gariepinus* (**A**) and *Oreochromis amphimelas* (**B**) from Lake Manyara

Samples of African catfish and tilapia (*Clarias gariepinus and Oreochromis amphimelas* respectively) were collected in April 2022 from five fish landing sites in Lake Manyara as shown in Fig. 1. All fish from the lake are unloaded at these landing sites and therefore these locations are the primary sources of fish for the local and outside markets. Ten samples of African catfish (Fig. 2A) were collected from each sampling point regardless of their size in order to represent what is available for consumption by the public. For tilapia (Fig. 2B), samples were collected by weight (9–10 kg) from each sampling point because of their small body size. Samples were kept in cool boxes and transferred to the Tanzania Atomic Energy Commission (TAEC) Laboratories in Arusha for preparation to obtain edible tissues (muscle and skin), where they were rinsed with distilled water (EPA 2000) and dissected to get to be analysed for trace elements. In this case, edible tissues from all the collected fish were kept separate per fish type and mixed into composite samples per sampling site. All utensils were cleaned according to the procedure described in (EPA 2000) using detergents, tap water, distilled water and 0.5% nitric acid prior to their use in sample preparation. Composite samples were dried in an oven at 60°C for 72 h then grounded using mortar and pestle. Grounded samples were stored in desiccators or plastic zip bags to avoid of moisture absorption as described in Sawe et al. (2019).

Sediment samples from Lake Manyara were collected using simple surface grabs of the top 5 cm on 44 locations in four different areas of the Lake (Fig. 1): northeast, northwest, southeast and southwest (Wynants et al. 2020). While a gridded approach to sediment sampling would be more appropriate (Bai et al. 2011), this was logistically not possible due to the large size of the lake and unsuitability of the sodic water for outboard engines.

Each samples stored in desiccators or plastic zip bags was divided into three portions of around 4 g. Each portion was mixed with around 0.9 g of the binding material, highly homogenized, and pressed in pellet form with a diameter of about 32 mm. The pellets were analyzed using Energy Dispersive X-Ray Fluorescence (EDXRF) spectrometry system utilizing X-Lab Pro™ software. Total elemental concentrations were estimated using the relation below (Rousseau et al. 1996).

$$ C_{E} = \left( {K_{E} \times I_{E} \times M_{E} } \right) $$

where *C*_E_ = elemental concentration (e.g. mg kg^–1^) of element ‘E’; *K*_E_ = the calibration constant for element ‘E’ (%/kcps); I_E_ = net peak intensity for analyte element ‘E’ (kcps) and *M*_E_ = matrix correction term for element ‘E’. The calibration constant is established using a standard sample.

The analytical accuracy was verified by adding a material of known elemental concentrations (same matrix) to every set of fish samples analyzed on the EDXF. Slight deviation (s) from expected values were adjusted to ensure validity of reported results. The certified reference material (IAEA–436)—trace elements and methyl mercury in tuna fish flesh homogenate (IAEA 2006) was used during this study.

The established amounts of potentially toxic metals in fish were compared with FAO/WHO permissible levels (mg.kg^−1^) and FAO/WHO Provisional Table Weekly Intake (PTWI), per kg of body weight of approximately 70 kg (see Table 1). According to WHO (2021), “*PTWI is* *an estimate of the amount of a substance in air, food, soil or drinking water that can be assimilated weekly per unit body weight (bw) over a lifetime without causing considerable health risk*”.

**Table 1:** Permissible levels of metals in fish

Element	Permissible level (mg.kg^−1^)	PTWI (mg/person/week)	References
Zinc	100	490	WHO (1989); Nauen (1983)
Cu	30	245	WHO (1989); Nauen (1983); Solgi (2019)
Pb	1.5	1.75	WHO (1989); Nauen (1983); Joint Expert Committee for Food Additives (2003); Ishak et al (2020)
Ni	–	2.45	WHO (1989); Nauen (1983)

Collected sediments were sieved to < 63 µm and analysed by Wave Length Dispersive X-Ray Fluorescence (WD-XRF, PANalytical Axios Max; OMNIAN application) as pressed pellets in the University of Plymouth Consolidated Radioisotope Facility as discussed in Wynants et al. 2020.

The data was tested for normality using Shapiro–Wilk test, where Ni and Cu were found to be non-normally distributed p ˃ 0.05 (0.09 and 0.11, respectively), while Zn and Pb were normally distributed p ˂ 0.05 (0.001 and 0.008) respectively. The non-parametric Kruskal Wallis test was carried out in the Statistical Package for Social Science (SPSS) to test if there were significant differences in the analytical concentrations measured between different sites and different species. A p-value less than 0.05 (*p* < 0.05) was considered statistically significant and vice versa. Sediment PTM concentrations were compared between the different lake areas to evaluate the difference in exposure levels (Bai et al. 2011).

## Results and Discussion

The concentrations of potentially toxic elements in fish from Lake Manyara Northern Tanzania are presented in Tables 2 and 3. Results are reported as minimum (min), maximum (max), and mean values with their corresponding standard deviations (SD) and are presented in mg kg^−1^ dry weight (dw). The elements covered in this study are copper (Cu), nickel (Ni), lead (Pb), and zinc (Zn). From Tables 2 and 3, it is evident that the maximum level of Cu (16.91 mg kg^−1^) was measured in African catfish sampled from Oltukai and the lowest level (5.73 mg kg^−1^) was measured in African catfish sampled from Minjingu. Cu levels in fish from literature have been reported as ranging from 0.7 to 27.0 mg kg^–1^ in muscles of fish (Tapia et al. 2006), 254 mg kg^–1^ in the livers of fish (Fernandes et al. 2007), 1.65–9.17 ng g^–1^ in muscles of fish (Mziray and Kimirei 2016). The obtained results were within the maximum allowable level of 30 mg kg^–1^ (WHO 1989).

**Table 2:** Minimum, maximum, and mean ± standard deviations (SD) of elemental concentrations (mg kg–1) in Tilapia (*Oreochromis amphimelas*) from Lake Manyara, Northern Tanzania

Location	Fish name and quantity	Elemental concentrations											
		Ni			Cu			Zn			Pb		
		Min	Max	Mean ± SD	Min	Max	Mean ± SD	Min	Max	Mean ± SD	Min	Max	Mean ± SD
Jangwani ziwani	Tilapia (10 kg)	0.70	0.89	0.80 ± 0.09	10.33	15.5	12.69 ± 1.93	40.22	50.5	44.63 ± 2.98	0.55	0.68	0.65 ± 0.01
Migunga mitatu	Tilapia	0.64	0.83	0.68 ± 0.08	11.24	14.31	12.89 ± 0.89	39.73	52.80	43.80 ± 2.58	0.40	0.68	0.58 ± 0.10
Oltukai	Tilapia	0.66	0.82	0.88 ± 0.05	6.85	16.00	14.79 ± 1.61	38.42	45.81	41.73 ± 1.78	0.63	0.77	0.73 ± 0.01
Minjingu	Tilapia	0.62	0.85	0.82 ± 0.12	6.49	12.84	8.92 ± 1.31	34.31	42.83	35.90 ± 4.44	0.44	0.76	0.60 ± 0.06
Mfulo wa Ng’ombe	Tilapia (10 kg)	0.74	0.80	0.75 ± 0.08	5.98	10.25	7.14 ± 1.61	33.99	36.26	34.58 ± 1.10	0.56	0.70	0.65 ± 0.01
OVERALL MEAN				0.79			11.29			40.13			0.64

**Table 3:** Minimum, maximum, and mean ± standard deviations (SD) of elemental concentrations (mg kg–1) in African catfish (Clarias gariepinus) from Lake Manyara, Northern Tanzania

Location	Fish name and quantity	Elemental concentrations											
		Ni			Cu			Zn			Pb		
		Min	Max	Mean ± SD	Min	Max	Mean ± SD	Min	Max	Mean ± SD	Min	Max	Mean ± SD
Jangwani ziwani	African catfish (n = 10)	0.63	0.82	0.75 ± 0.10	8.22	11.65	10.09 ± 2.13	38.5	47.21	40.43 ± 1.23	0.43	0.64	0.50 ± 0.01
Migunga mitatu	African catfish (n = 10)	0.65	0.78	0.76 ± 0.08	5.84	15.03	9.51 ± 2.27	33.84	41.44	35.84 ± 2.04	0.41	0.72	0.64 ± 0.01
Oltukai	African catfish (n = 10)	0.74	0.91	0.91 ± 0.13	8.93	16.91	14.72 ± 1.33	37.55	51.11	47.10 ± 3.10	0.53	0.67	0.65 ± 0.03
Minjingu	African catfish (n = 10)	0.64	0.88	0.77 ± 0.14	5.73	9.63	5.73 ± 1.46	35.33	48.23	37.90 ± 2.14	0.38	0.57	0.48 ± 0.08
Mfulo wa Ng’ombe	African catfish (n = 10)	0.68	0.90	0.80 ± 0.05	7.6	12.97	10.00 ± 2.47	42.31	46.18	43.45 ± 2.23	0.72	0.81	0.73 ± 0.01
OVERALL MEAN				0.80			10.45			42.22			0.60

African catfish samples taken at Mfulo wa Ng’ombe and Minjingu had respectively the highest (0.81 mg kg^−1^) and lowest (0.38 mg kg^−^1) concentrations of lead (Table 3). These values are within the ranges of lead concentrations for fish muscles from the Black and Aegean seas reported in the literature between 0.33 and 0.93 mg kg^−1^ (Uluozlu et al. 2007), 0.01 to 0.15 mg kg^−1^ (dw) for fish muscles from the Ria de Averio in Portugal (Perez et al. 2001), 71 to 278 μg kg^−1^ (dw) for fish muscles from the Eastern Aegean Sea (Uluozlu et al. 2007). The results of this study were within permissible limits, and the maximum allowed level of lead in fish is 1.50 mg kg^−1^ (Joint Expert Committee for Food Additives 2003), Ishak et al. 2020).

As shown in Tables 2 and 3, African catfish samples taken from Oltukai station had the highest Ni concentration (0.91 mg kg^−1^) and tilapia samples taken from Minjingu station had the lowest (0.62 mg kg^−1^). According to reports, fish muscle tissue from Indian markets have Ni concentrations between 0.03 and 1.38 mg kg^−1^ (Sivaperumal et al. 2007), between 0.12 and 0.15 mg kg^−1^ in coastal Tanzania (Mziray and Kimirei 2016), and between 0.06 and 1.59 mg kg^−1^ in the Mediterranean Sea (Turkmen et al. 2008). Maximum Ni concentrations in fish are not known, but the WHO has set the Provisional Table Weekly Intake (PTWI) at 2.45 mg person^−1^ week^−1^ kg of body weight^−1^. Basing on the highest concentration of Ni in African catfish (0.91 mg kg^−1^), a person could eat approximately 2.7 kg of fish muscle tissue from Manyara in a week. It would be 3.1 kg based on the overall mean in African catfish (0.80 mg kg^−1^). Given these high masses, it is unlikely that the Ni concentrations in fish from Lake Manyara pose a risk to human’s health.

The highest level of Zn (52.80 mg kg^–1^) was measured in tilapia sampled at Migunga mitatu and the lowest (33.84 mg kg^–1^) was measured in African catfish also sampled at Migunga mitatu (Table 3). It was interesting to note that the highest and lowest levels of Zn were measured at the same location and in different fish species, especially considering that the average value of Zn is higher in African catfish than tilapia. The reason for this observation is not immediately clear. Levels of Zn reported for fish in other parts of the world are in the range of 1.35–6.69 mg kg^−1^ in muscle, 2.71–78.70 mg kg^−1^ in liver, and 7.27–16.87 mg kg^−1^ in gonads (Uluturhan and Kucuksezgin 2007). The obtained results were within the maximum allowable levels of Zn which is 100 mg kg^–1^ for fish (Nauen 1983, WHO 1989).

In this study, the overall mean concentrations of metals in both fish species together (not shown in the tables) were found to be 41.18, 10.87, 0.80 and 0.62 mgkg^−1^ for Zn, Cu, Ni and Pb, respectively. This suggest a trend of Zn > Cu > Ni > Pb for the two fish species combined. Looking at the species separately, tilapia had average concentrations of 0.79, 11.29, 40.13 and 0.64 mg kg^−1^ for Ni, Cu, Zn, and Pb, respectively (Table 2). African catfish showed an average concentration of 0.80, 10.45, 42.22 and 0.60 mgkg^−1^ for nickel, copper, zinc and lead, respectively (Table 3). The elemental concentrations in both tilapia and African catfish followed the trend of Zn > Cu > Ni > Pb. This trend suggest essential elements had higher concentrations than the non-essential element (Pb). The trend may endorse the essential biological roles of Zn, Cu and Ni in fish (Chen and Chen 2001, Bahnasawy et al. 2009). The results further showed that the concentrations of Cu and Pb were slightly higher in tilapia, while Ni and Zn were slightly higher in African catfish. These slight differences could be explained due to the specific feeding behaviour or differences in metabolic pathways. Evidenced by the small standard deviation, the variation in PTM concentration within the species was relatively low. The remaining variance can be explained by differences in life stage, size of the fish, and catchment characteristics of the dominant contributing tributary. This study focused on fish available in the market regardless of their size. However, further research on differentiating PTM concentrations in different fish sizes and organs is desirable since it could form an additional indication if metals are accumulating when fish are growing or in certain parts of fish.

Variations of elemental concentrations between fish types investigated by Kruskal–Wallis test revealed that there were no significant differences in Nickel (*p* = 0.40), Lead (*p* = 0.40), Copper (*p* = 0.30) and Zinc (*p* = 0.34) concentrations across categories of tilapia and African catfish. Since no significant higher values of metals were found in the omnivorous African catfish, we can infer that the investigated PTMs are not accumulating higher up the food chain in Lake Manyara, allowing us to reject this hypothesis. Moreover, there was no difference in the PTM variance within the species, which was low in both species. Since the fish population in Lake Manyara supports a large number and diversity of fish-eating avifauna (Janssens de Bisthoven et al. 2020), it is unlikely these are negatively impacted by the studied potential toxic metals. However, further investigation of PTM concentrations in the fish-eating avifauna, suspended materials, and in the liver and kidneys of fish is needed to validate this finding. Moreover, a better understanding of the Lake Manyara foodweb is needed for a holistic overview of the potential impacts of PTMs on the entire ecosystem (Lemmens et al. 2017).

The analysis of PTMs in the sediment samples (Table 4) revealed significant differences in their concentrations between different areas in the lake. The concentration of Ni in the sediments varied significantly (*p* < 0.01), with higher values in the north. Sediment Pb also varied significantly (*p* < 0.01) with highest concentrations in the southwest. Zn concentrations in the sediments also significantly differed (p-value < 0.01) with the highest values in the northeast. Only the Cu concentrations did not significantly differ in lake sediments (*p* = 0.32). However, it is important to note the high variance in Cu concentration driven by some high outlier values. These high differences in PTM concentrations in the sediments are most likely caused by natural differences in the geochemistry of the contributing tributaries (Wynants et al. 2020), although unnaturally high Cu concentrations have been observed in soils under coffee plantations located in the Makuyuni catchment, most likely due to fungicide applications (Wynants et al. 2021a). However, regardless of these differences in sediment chemistry, there were no significant differences in the fish muscle concentrations of Nickel (*p* = 0.29), Copper (*p* = 0.29), Zinc (*p* = 0.33), and Lead (*p* = 0.35) between the different landing sites. If increased mobilisation of metals from mining or erosion in the wider catchment would cause increased uptake of PTMs in fish, we would expect these concentrations to be higher in the fish landing sites near the main river inlets and the hotspot PTM concentration areas (Bai et al. 2011). The lack of significant differences between the fish landing sites indicate that we can reject this hypothesis. The concentration of PTMs in the fish were also an order of magnitude lower compared to those in the sediments, indicating that they are not impacted by PTMs entering the lake as sediments. This is likely because the PTMs remain bound to the sediment particles and are relatively immobile in the alkaline environment of Lake Manyara (Kicińska et al. 2022). However, additional analyses are needed on the water chemistry of Lake Manyara to gain a better understanding of PTM mobility in its total environment. In this study, the fish were gathered in April nearing the end of the rainy season. However, it is possible that when the lake dries, the concentration of PTMs will increase, potentially affecting the concentrations in the fish tissues. A promising area of further study is to monitor potential increases in PTM concentrations throughout the fishing season.

**Table 4:** Minimum, maximum, and mean ± standard deviations (SD) of elemental concentrations (mg kg–1) in sediment from Lake Manyara, Northern Tanzania

Location	Elemental concentrations															
	Ni				Cu				Zn				Pb			
	Min	Max	Mean ± SD	Median	Min	Max	Mean ± SD	Median	Min	Max	Mean ± SD	Median	Min	Max	Mean ± SD	Median
Northeast	93.3	148.1	112.3 ± 15.1	111.3	24.7	114.8	79.0 ± 20.4	79.4	122.9	175.0	149.5 ± 14.8	147.6	24.7	49.1	36.4 ± 7.8	36.9
Northwest	116.8	129.4	124.7 ± 4.0	124.1	73.4	87.0	78.8 ± 4.2	78.4	88.0	142.8	126.6 ± 18.4	136.2	24.7	36.6	30.4 ± 3.8	28.7
Southeast	61.0	119.9	90.8 ± 18.1	91.2	49.3	386.3	133.5 ± 130.7	69.9	73.5	142.7	108.2 ± 19.2	104.6	8.1	40.6	18.1 ± 10.0	18.6
Southwest	84.1	138.2	112.5 ± 15.8	109.6	57.3	428.3	132.6 ± 121.7	93.9	99.3	183.8	144.7 ± 27.1	138.1	24.7	36.6	30.4 ± 3.8	38.6
OVERALL MEAN			107.2				106.1				131.1				31.1	

Lack of fish consumption data of the human population in the study area and in Tanzania as a whole prevented the assessment of PTM intake due to fish consumption. However, using the WHO’s Provisional Table Weekly Intake (PTWI) of metals, we developed the first recommendation of the amount of Lake Manyara fish that can be consumed without appreciable health risk. Based on the highest concentration of each metal found in fish, one is recommended to consume a maximum of 14.5, 2.7, 2.2, and 9.3 kg of fish per week in order to reach the PTWI for Cu, Ni, Pb and Zn respectively. Since the 2.2 kg related to Pb concentration is the lowest number, this could act as a provisional guideline for fish consumption from Lake Manyara.

Levels of lead (Pb), zinc (Zn), copper (Cu) and nickel (Ni) in edible parts of tilapia and African catfish from Lake Manyara Northern Tanzania were assessed during this study. The highest levels of Nickel and copper were found in African catfish at the Oltukai site, while the highest levels of zinc were found in tilapia at Migunga mitatu site. The highest lead concentration was found in African catfish at Mfulo wa ng’ombe site. The results did not show any significant differences in PTM concentrations between Manyara tilapia and African catfish, nor between sampling points. This indicates that the metals are not accumulating in the food chain, nor are the fish near the river inlets more impacted, allowing us to reject these hypotheses. The trend of elemental concentrations in both tilapia and African catfish followed Zn > Cu > Ni > Pb and none of the analyzed elements exceeded the permissible levels recommended by the Food and Agriculture Organization of the United Nations (FAO). This indicates that the lake and the fish are not impacted by pollution of the studied metals.

Based on the Provisional Table Weekly Intake of metals of the World Health Organization (WHO) and the highest found concentration of Pb in fish, we recommend a maximum consumption of 2.2 kg fish muscle tissue per week from Lake Manyara. Given this relatively high value, these results suggest that it is unlikely that the analyzed PTMs form a health risk to the fish consumers in Tanzania. Based on the results of this study, it can be concluded that fish from Lake Manyara are safe for human consumption provided that the amount consumed does not exceed estimated values based on PTWI. Nonetheless, continued monitoring of heavy metals in fish from Lake Manyara is recommended because of the increasing human activities in the catchment area.
